# Mxi1-0 Promotes Hypoxic Pulmonary Hypertension *Via* ERK/c-Myc-dependent Proliferation of Arterial Smooth Muscle Cells

**DOI:** 10.3389/fgene.2022.810157

**Published:** 2022-03-23

**Authors:** Liang Dong, Xinning Liu, Bo Wu, Chengwei Li, Xiaomin Wei, Gulinuer Wumaier, Xiujuan Zhang, Jing Wang, Jingwen Xia, Yuanyuan Zhang, Ruzetuoheti Yiminniyaze, Ning Zhu, Jing Li, Daibing Zhou, Youzhi Zhang, Shuanghui Li, Junzhu Lv, Shengqing Li

**Affiliations:** ^1^ Department of Pulmonary and Critical Care Medicine, Huashan Hospital, Fudan University, Shanghai, China; ^2^ Department of Lung Transplantation, Wuxi People’s Hospital, Wuxi, China

**Keywords:** hypoxic pulmonary hypertension (HPH), max interacting protein 1–0 (Mxi1-0), pulmonary arterial smooth muscle cells (PASMCs), cell proliferation, MEK/ERK, c-Myc

## Abstract

**Background:** Hypoxic pulmonary hypertension (HPH) is a challenging lung arterial disorder with remarkably high incidence and mortality, and so far patients have failed to benefit from therapeutics clinically available. Max interacting protein 1–0 (Mxi1-0) is one of the functional isoforms of Mxi1. Although it also binds to Max, Mxi1-0, unlike other Mxi1 isoforms, cannot antagonize the oncoprotein c-Myc because of its unique proline rich domain (PRD). While Mxi1-0 was reported to promote cell proliferation *via* largely uncharacterized mechanisms, it is unknown whether and how it plays a role in the pathogenesis of HPH.

**Methods:** GEO database was used to screen for genes involved in HPH development, and the candidate players were validated through examination of gene expression in clinical HPH specimens. The effect of candidate gene knockdown or overexpression on cultured pulmonary arterial cells, e.g., pulmonary arterial smooth muscle cells (PASMCs), was then investigated. The signal pathway(s) underlying the regulatory role of the candidate gene in HPH pathogenesis was probed, and the outcome of targeting the aforementioned signaling was evaluated using an HPH rat model.

**Results:** Mxi1 was significantly upregulated in the PASMCs of HPH patients. As the main effector isoform responding to hypoxia, Mxi1-0 functions in HPH to promote PASMCs proliferation. Mechanistically, Mxi1-0 improved the expression of the proto-oncogene c-Myc *via* activation of the MEK/ERK pathway. Consistently, both a MEK inhibitor, PD98059, and a c-Myc inhibitor, 10058F4, could counteract Mxi1-0-induced PASMCs proliferation. In addition, targeting the MEK/ERK signaling significantly suppressed the development of HPH in rats.

**Conclusion:** Mxi1-0 potentiates HPH pathogenesis through MEK/ERK/c-Myc-mediated proliferation of PASMCs, suggesting its applicability in targeted treatment and prognostic assessment of clinical HPH.

## Introduction

Hypoxic pulmonary hypertension (HPH) due to lung diseases and/or chronic hypoxia is among the most common groups of pulmonary hypertension with high mortality (Badesch et al., 2010; Galiè et al., 2015). Although the two key pathological processes, vasoconstriction and pulmonary vascular remodeling, have been adequately investigated (Dunham-Snary et al., 2017; Woo et al., 2019; Young et al., 2019), the 3 years survival rate of HPH is significantly worse than of other categories of HPH (Hurdman et al., 2012). In advanced lung disease, regional hypoxic vasoconstriction improves the matching of perfusion and alveolar ventilation, but subsequently leads to the increase in pulmonary circulation pressure (Naeije and Dedobbeleer, 2013; Li et al., 2018), underlining the difficulty of HPH treatment despite the availability of novel drugs (Prins et al., 2017; Cassady and Reed, 2019). Therefore, there is an urgent need to develop novel therapeutics based on the identification of key players in the pathogenesis of HPH. In particular, although hypoxia-induced cell proliferation of vascular endothelial and smooth muscle cells is a hallmark of HPH, the underlying mechanisms remain largely elusive.

Max interacting protein 1–0 (Mxi1-0) is an alternative transcript of Mxi1 involved in the Myc-Max-Mad network (Zervos et al., 1993; Dugast-Darzacq et al., 2004). Mxi1-0 shares exons 2 to 6 with the originally cloned Mxi1 (here-in-after referred to as Mxi1-1). The common basic region helix-loop-helix/leucine zipper (bHLH/LZ) enables Mxi1-0 to bind to Max and regulate cell behavior (Hurlin and Huang, 2006). Compared with Mxi1-1, Mxi1-0 has an alternative first exon (exon 0), encoding a different SIN3 interaction domain (SID) (Engstrom et al., 2004), and is reported to promote proliferation of various types of cells, such as endothelial cells and neuroblastoma cells (Armstrong et al., 2013; Wu et al., 2017). However, it is uncharacterized whether and how Mxi1-0 plays a regulatory role in HPH-related over-proliferation of pulmonary arterial endothelial cells (PAECs) or pulmonary arterial smooth muscle cells (PASMCs).

In this study, we found in clinical specimens that Mxi1-0 was crucially involved in the development of HPH. Mxi1-0 but not Mxi1-1 improved the mitosis of PASMCs. Mechanistically, Mxi1-0 was transcriptionally activated by hypoxia and upregulates the proto-oncogene c-Myc *via* MEK/ERK signaling.

## Materials and Methods

### Sample Collection and Ethics Statement

6 chronic lung disease (CLD) samples with or without hypertension were obtained during lung transplantation, and three samples of donor lung tissues were taken from the lungs that were not transplanted. All experiments related to human samples were conducted in accordance with the Declaration of Helsinki and were approved by the ethics committee. All subjects provided written informed consent prior to participation in the study.

### Hematoxylin and Eosin Staining

The fixed lungs were sliced in the mid-sagittal plane, embedded in paraffin, and cut into 5 μm thick sections with a microtome. Then, the sections were placed on glass slides, stained with hematoxylin and eosin (HE) staining for morphological analysis, and visualized under an Olympus BX41 microscope (Tokyo, Japan).

### Immunohistochemistry and Immunofluorescence Staining

Paraffin-embedded lung tissue sections were deparaffinized in xylene and rehydrated in a graded ethanol series to PBS. Antigen retrieval was performed by pressure cooking in citrate buffer for 10 min. The sections were permeabilized by incubation with 0.3% Triton X-100 and blocked with 5% donkey serum albumin in a humidified chamber for 1h, and were immunostained with primary antibodies to Mxi1 (1:200, ab28740, Abcam Ltd., Cambridge, United Kingdom) or α-smooth muscle actin (α-SMA) (1:200, 48938, CST, Boston, United States). After overnight incubation, sections were washed and incubated with the respective secondary antibodies, donkey anti-rabbit IgG (1:1,000, Jackson Immuno, PA, United States), alexa 594 donkey anti-rabbit IgG (1:1,000, Jackson Immuno, PA, United States), or oralexa 488 donkey anti-mouse IgG (1:1,000, Jackson Immuno, PA, United States) for 1 h. For immunohistochemistry, sections were counterstained with hematoxylin and detected by incubation with the DAB substrate. For immunofluorescence staining, sections were counterstained with nuclear DAPI (1:1,000) and mounted with fluorescent mounting medium.

### Cell Culture and Transfection

Primary human PASMCs were purchased from ScienCell, Inc (#3110) and cultured in smooth muscle cell medium (SMCM, ScienCell). Primary human PAECs were purchased from ScienCell, Inc (#3100) and cultured in endothelial cell medium (ECM, ScienCell). Cells were used at passages 4–7. In the cell-growth assay, PASMCs were exposed to normoxia, hypoxia, and hypoxia plus various antagonists as indicated. Cells in the normoxia group were maintained at 37°C in 21% O_2_, 74% N_2_, and 5% CO_2_ (Forma 370, Thermo, United States). Cells in the hypoxia groups were separately cultured at 1% O_2_, 94% N_2_, and 5% CO_2_ (Forma 3131, Thermo, United States). When cells reached 80% confluence, they were transfected with different siRNAs using Lipofectamine 3000 Transfection Reagent (Invitrogen). 6 h after transfection, cells were cultured in serum containing medium for a resting period of 24 h, followed by hypoxia exposure for different times. Mxi1-1 siRNA (target sequence: 5′-CGUCGCACAUGUUCC GGAACG-3′) and Mxi1-0 siRNA (target sequence: 5′-CAGCGAGAACUCGAUGGAGAATT-3′) were synthesized by GenePharma (Shanghai Gene Pharma Co.,). As a control, commercially available non-targeting siRNA (si-Control) was used.

### Immunocytofluorescence Assay

Human PASMCs grown on chamber slides were fixed with 4% paraformaldehyde, permeabilized by incubation with 0.3% Triton X-100 and blocked with 5% donkey serum albumin for 2 h. Then, slides were immunostained overnight with primary antibodies to Mxi1 (1:100, ab28740, Abcam Ltd., Cambridge, United Kingdom), followed by 1 h incubation with a secondary antibody, alexa 594 donkey anti-rabbit IgG (1:200, Jackson Immuno, PA, United States). After incubation, slides were counterstained with nuclear DAPI (1:1,000) and mounted with fluorescent mounting medium. Fluorescent images were taken with fluorescence microscope (Olympus, Japan).

### Lentiviral Infection

The recombinant lentiviruses expressing Flag-tagged Mxi1-1 and HA-tagged Mxi1-0 were purchased from Genscript (Jiangsu, China). To prepare Mxi1-1 or Mxi1-0 overexpression viral particles, HEK293T cells were co-transfected with each viral vector and the packaging vectors (pMD2.0G and psPAX) using JetPEI purchased from Qbiogene (Montreal, Canada) following the manufacturer’s protocol. The medium was replaced 4 h after transfection, and cells were cultured for a further 36 h. Viral particles were harvested, filtered using a 0.45 µm syringe filter, and combined with 10 μg/ml polybrene (Sigma, MO, United States). PASMCs at 60% confluency were treated with these particles overnight. The culture medium was then replaced with fresh complete growth medium, and cells were cultured for a further 24 h and selected with puromycin (1 μg/ml). The selected cells were used in further experiments.

### Quantitative Real-Time PCR

The mRNA level was determined by qRT-PCR. Total RNA was extracted from cells or tissues using Trizol reagent (Invitrogen, CA, United States) according to the manufacturer’s protocol. RNA was subsequently reverse transcribed into cDNA using ReverTra Ace qPCR RT Kit (TOYOBO, Japan). cDNA was amplified and detected using Hieff qPCR SYBR Green Master Mix (Yeasen, Shanghai, China). The PCR primers were as follows: 5′-GGA​CCT​GAC​TGA​CTA​CCT​CAT-3′ and 5′-CGT​AGC​ACA​GCT​TCT​CCT​TAA​T-3′ for β-actin, 5′-GAG​GCT​GCC​GAG​TTT​TTG​G-3′ and 5′-TCGGCATGGACGGGAAT-3′ for Mxi1-1, and 5′-GAG​ACC​GAC​ACA​CAC​TCC​CAT​A-3′ and 5′-CGAAAAGCCGGCCTGACT-3′ for Mxi1-0. Fold change of RNA species was analyzed with the formula (2^−ΔΔCt^), and was normalized to β-actin expression.

### Western Blotting

Total lysates of cells or tissues were homogenized in RIPA lysis buffer (Beyotime, Shanghai, China) supplemented with a protease inhibitor, phenylmethyl sulfonyl fluoride (1 mM). Equivalent amounts of protein were separated by SDS- polyacrylamide gels and transferred to 0.22 μM nitrocellulose membranes (Millipore, MA, United States). After blocking, the membranes were probed with one of the following primary antibodies overnight at 4°C: anti-Mxi1 (1:1,000, sc-130627, Santa Cruz, TX, United States), anti-MEK (1:1,000, 4,694, CST, Boston, United States), anti-phospho-MEK (1:1,000, 9,154, CST, Boston, United States), anti-ERK (1:1,000, 4,695, CST, Boston, United States), anti-phospho-ERK (1:1,000, 4,370, CST, Boston, United States) and anti-c-Myc (1:1,000, 13987, CST, Boston, United States). Then, membranes were incubated with secondary antibodies for 1 h at room temperature. Bound antibodies were detected using a Super ECL Detection Reagent (Yeasen, Shanghai, China) and imaged on a Tanon Western blotting detection system (Tanon, Shanghai, China).

### Cell Viability Assay

Cells were seeded into 96-well plates at a density of 2000/well and incubated with either vehicles or inhibitors. After exposure to nomorxia or hypoxia, a total of 110 μL of DMEM containing the CCK-8 solution (Beyotime, Shanghai, China) [CCK8:DMEM (v/v) = 1:10] was added to each well, and cells were incubated for 4 h. Finally, cell viability was determined by measuring the absorbance at 450 nm using a multi-well spectrophotometer (Bio-RAD, CA, United States).

### Cell Proliferation Assay

Proliferation of cells was detected with a BeyoClickEdU Cell Proliferation Kit with Alexa Fluor 647 (Beyotime, Shanghai, China) following the manufacturer’s protocol. Briefly, cells were seeded into 12-well plates at a density of 10000/well and incubated with siRNA or vehicle. After exposure to nomorxia or hypoxia for 48h, cells were treated with EdU (20 µm) for 3 h and subjected to fixing and permeabilization. Then, cells were exposed in click additive solution for 30 min followed by Hoechst staining for 10min, and finally observed with fluorescence microscope (Olympus, Japan).

### Animal Models

Adult male Sprague-Dawley rats weighing 150–200 g were purchased from the Slake Company (Shanghai, China). All protocols and surgical procedures were approved by the Fudan University Veterinary Medicine Animal Care and Use Committee. Animals were randomly divided into five groups (*n* = 5/group): 1) normoxia (Nor), 2) chronic hypoxia (Hyp), 3) chronic hypoxia and treatment with DMSO (DMSO), 4) chronic hypoxia and treatment with 0.15 mg/kg/tiw PD98059 (PD0.15), and 5) chronic hypoxia and treatment with 0.3 mg/kg/tiw PD98059 (PD0.3). PD98059 was administered *via* intraperitoneal injection. Rats in the normoxia group were housed at ambient barometric pressure for 28 days (-718 mmHg, PO_2_ maintained in 150.6 mmHg). Rats in the hypoxia groups were housed in a hypobaric hypoxia chamber depressurized to 380 mmHg (PO_2_ in 79.6 mmHg) for 8 h/day for 28 days as previously described (Li et al., 2019). All animals were raised under a 12 h:12 h light-dark cycle and were freely supplied food and water. The room temperature was maintained at 25°C, and the bedding was changed once per week.

### Echocardiography and Hemodynamic Analysis

After 28 days, rats were fasted overnight and initially anesthetized with isoflurane inhalation. Echocardiography was performed with Visual Sonics Vevo 2100 ultrasoundmachine and 12S rodent probe (GE Healthcare, CT, United States) to determine pulmonary artery acceleration time (PAAT) and tricuspid annulusplain systolic excursion (TAPSE). Data were analyzed with EchoPAC software (GE Healthcare, CT, United States). Then, haemodynamic analysis was performed as previously described (Xia et al., 2018). Briefly, anesthesia was given to rats with 20% ethylurethanm *via* injecting intraperitoneally (4 ml/kg). After intubation, right ventricular systolic pressure (RVSP) was recorded. To investigate right ventricular hypertrophy (RVH), the right ventricle (RV), the left ventricle plus septum (LV + S) and the body weight (BW) were weighed, and the RV/(LV + S) ratio and RV/BW ratio were determined.

### Statistical Analysis

ImageJ software was used to scan the gray level of Western blotting images, and spss24.0 software was used for data statistical analysis. Statistical significance was assessed by comparing mean (±SD) values with Student’s t-test for independent groups. *p* < 0.05 was considered as statistically significant. The data were plotted with graphpad prism 8.0 software.

## Results

### Mxi1 Is Involved in the Clinical Development of Hypoxic Pulmonary Hypertension

**FIGURE 1:**
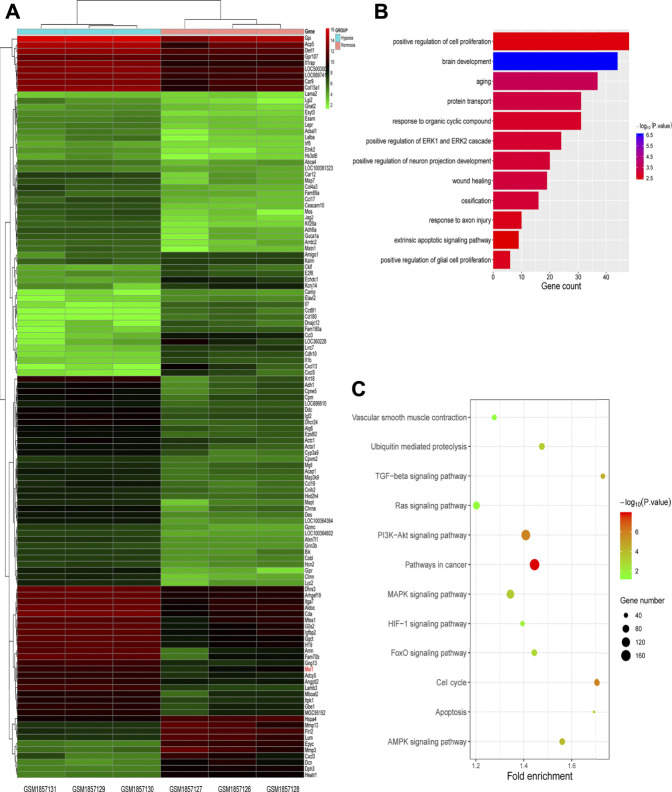
Screening for hypoxic pulmonary hypertension (HPH)-related genes. **(A)** Heatmap of genes differentially expressed in normal and hypoxic pulmonary arterial smooth muscle cells (PASMCs) in GEO database (dataset: GSM1857126∼1857131). **(B,C)** GO **(B)** and KEGG **(C)** enrichment analyses for differentially expressed genes described in **(A)**.

To identify candidate players in HPH pathogenesis, we first screened for genes differentially expressed between normal and hypoxic PASMCs using the GEO database (Figure 1A, dataset: GSM1857126-1857131). As a result, we obtained 602 genes, of which 200 were up-regulated and 402 were down-regulated in hypoxia samples. GO and KEGG enrichment analyses were performed using DAVID 6.8 database. The GO results indicated that genes were significantly enriched in cell proliferation, aging and response to organic cyclic compound, mainly concentrated in biological processes related to proliferation (Figure 1B), whereas KEGG analysis demonstrated that genes were mainly enriched in growth and differentiation related pathways, such as Ras signaling pathway, PI3K-Akt signaling pathway, MAPK signaling pathway, FOXO signaling pathway, cell cycle and AMPK signaling pathway (Figure 1C).

**FIGURE 2:**
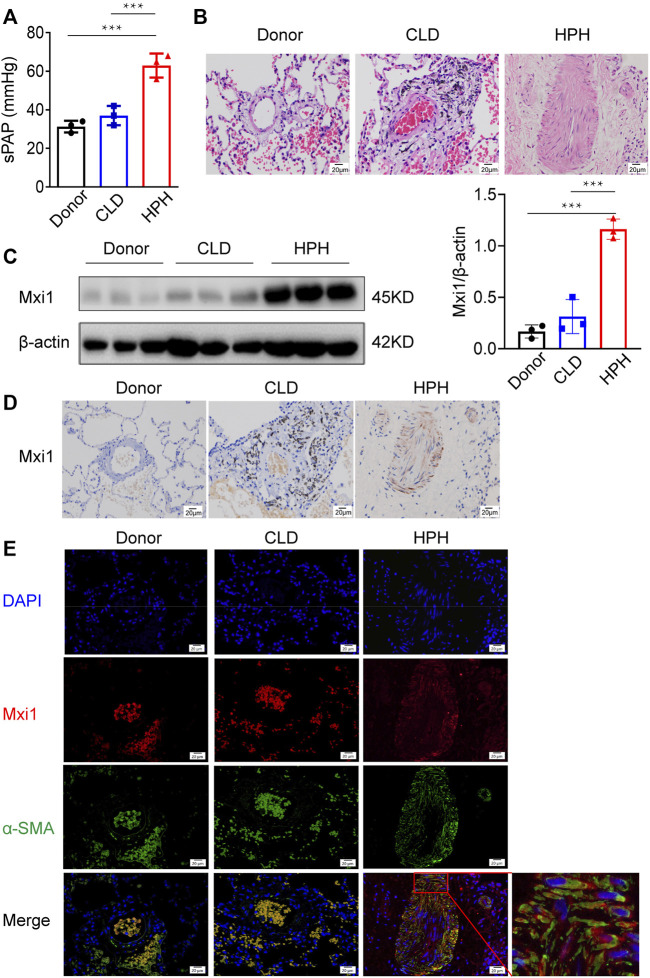
Mxi1 is upregulated in PASMCs of HPH patients. Lung samples of three donors, three chronic lung disease (CLD) patients and three hypoxic pulmonary hypertension (HPH) patients were collected and sectioned. **(A)** Hemodynamic analysis of patients before collection of samples. **(B)** Hematoxylin and eosin staining of paraffin-fixed lung sections was used for morphological analysis of pulmonary arteries. **(C)** The expression of Mxi1 was examined *via* Western blotting analysis, followed by quantification of the blots through densitometry and normalization to β-actin. **(D)** Representative paraffin lung tissue sections from donors, CLD, and HPH patients were subjected to immunohistochemical staining of Mxi1. **(E)** Representative sections in all groups were also subjected to immunofluorescence staining of Mxi1 and α-SMA with nuclei counterstained by DAPI (blue), and the image of HPH merge was zoomed. Scale bar, 20 μm. Data are shown as means ± SDs. For statistical significance, ^***^represents *p <* 0.001 compared with HPH patients.

Among the most significantly changed genes in HPH is Mxi1, which is remarkably upregulated in HPH and established to participate in the regulation of cell division (Armstrong et al., 2013; Wu et al., 2017). We next investigated the expression of Mxi1 in clinical HPH samples. Specimens of nine patients, including 3 with CLD but not hypertension, 3 with HPH and three donors of normal lungs, were collected and examined (Figures 2A,B). Western blotting analysis indicated that Mxi1 was significantly higher in pulmonary of HPH tissues than in those of CLD patients or donors, suggesting a correlation between Mxi1 expression and HPH occurrence (Figure 2C). Immunohistochemical staining of the lung tissues of HPH patients demonstrated specific upregulation of Mxi1 in the medial layer, consisting mainly of PASMCs (Figure 2D). Consistently, immunofluorescent staining showed that Mxi1 co-localized with α-SMA (Figure 2E). Thus, Mxi1 expression is induced in PASMCs during the development of HPH.

### Hypoxia Induces Expression of Mxi1-0 but Not Mxi1-1 in Pulmonary Arterial Smooth Muscle Cells

**FIGURE 3:**
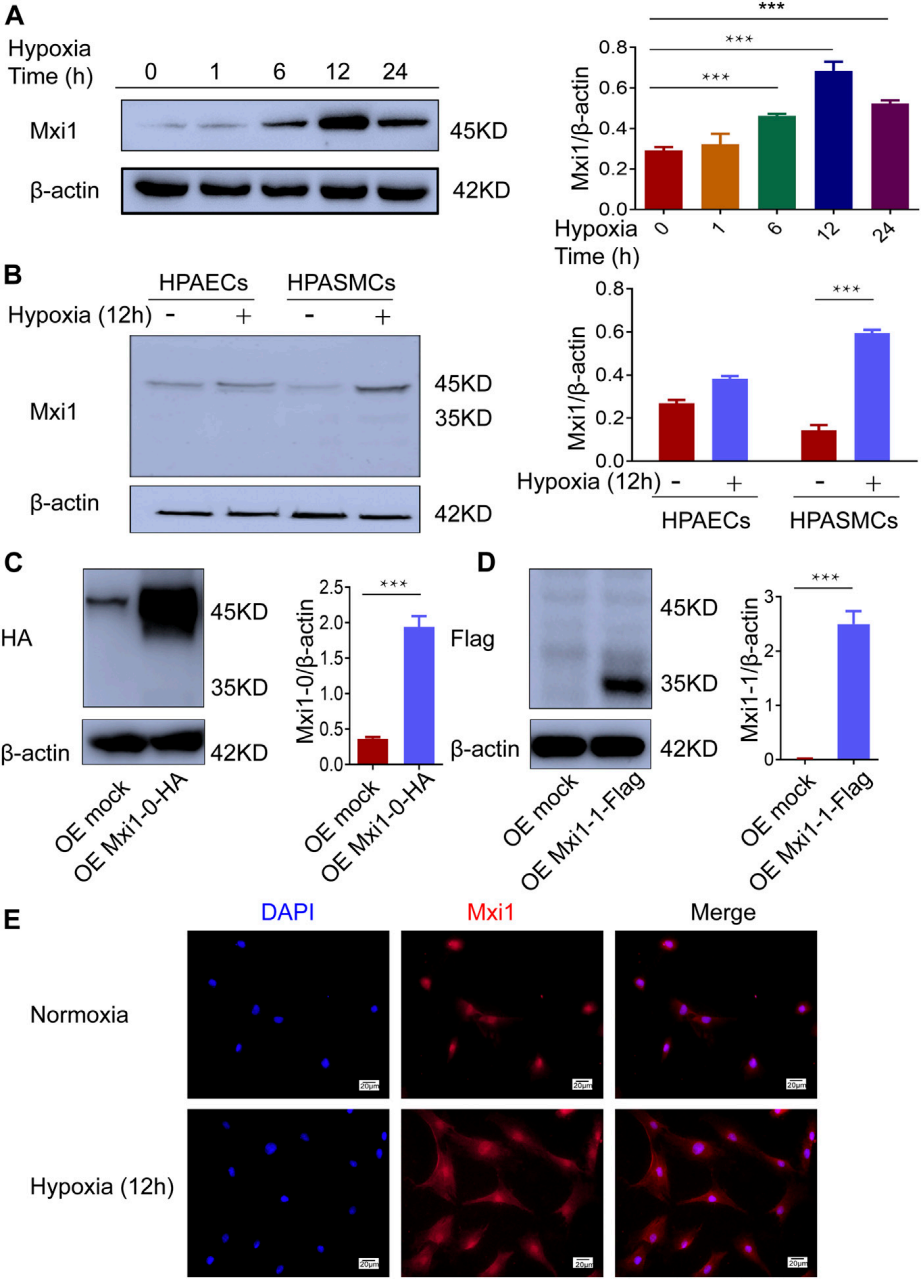
Hypoxia induces expression of Mxi1-0 but not Mxi1-1 in PASMCs. **(A)** PASMCs were exposed to hypoxia (1% O_2_) for indicated periods of time. Cell lysates were prepared and subjected to Western blotting assay. **(B)** Pulmonary arterial endothelial cells (PAECs) and PASMCs were exposed to normoxia (21% O_2_) or hypoxia for 12 h, and were subjected to Western blottinganalyses. **(C,D)** PASMCs were transfected with constructs for HA-tagged Mxi1-0 **(C)** or Flag-tagged Mxi1-1 **(D)**, and were subjected to Western blotting analyses. **(E)** PASMCs were exposed to normoxia or hypoxia for 12 h, and were subjected to immunostaining with a Mxi1 antibody and counterstaining of the nuclei with DAPI. Scale bar, 20 μm. Data from three independent experiments are shown as means ± SDs. For statistical significance, ^***^represents *p <* 0.001 compared to normoxia or the mock-transfected group.

We next explored the expression of Mxi1 isoforms in pulmonary arterial cells when exposed to hypoxia. Western blotting analysis showed that Mxi1 was induced by hypoxia in a time-dependent manner and peaked when exposed to hypoxic conditions for 12 h (Figure 3A). Hypoxia significantly upregulated Mxi1 in PASMCs but not PAECs (Figure 3B). Consistent with the assays using clinical pulmonary specimens (Figure 2C), we detected a 45 kD protein using the pan-Mxi1 antibody (Figure 3B). In addition, to determine the major isoforms induced by hypoxia, we generated vectors for tagged Mxi1-0 and Mxi1-1, and ectopically overexpressed these proteins in PASMCs. Western blotting analyses using antibodies recognizing the tag sequence detected 2 proteins with distinct molecular weights (Figures 3C,D), suggesting that Mxi1-0 was the predominant isoform upregulated by hypoxia (Figure 3A). Unlike Mxi1-1 that was reported to exhibit a predominant nuclear localization (Engstrom et al., 2004; Erichsen et al., 2015), Mxi1-0 resided both in the cytoplasma and the nucleus (Figure 3E). These findings strongly suggest that Mxi1-0 is remarkably upregulated in PASMCs when exposed to hypoxic conditions.

### Mxi1-0 but Not Mxi1-1 Mediates Hypoxia-Induced Pulmonary Arterial Smooth Muscle Cells Proliferation

**FIGURE 4:**
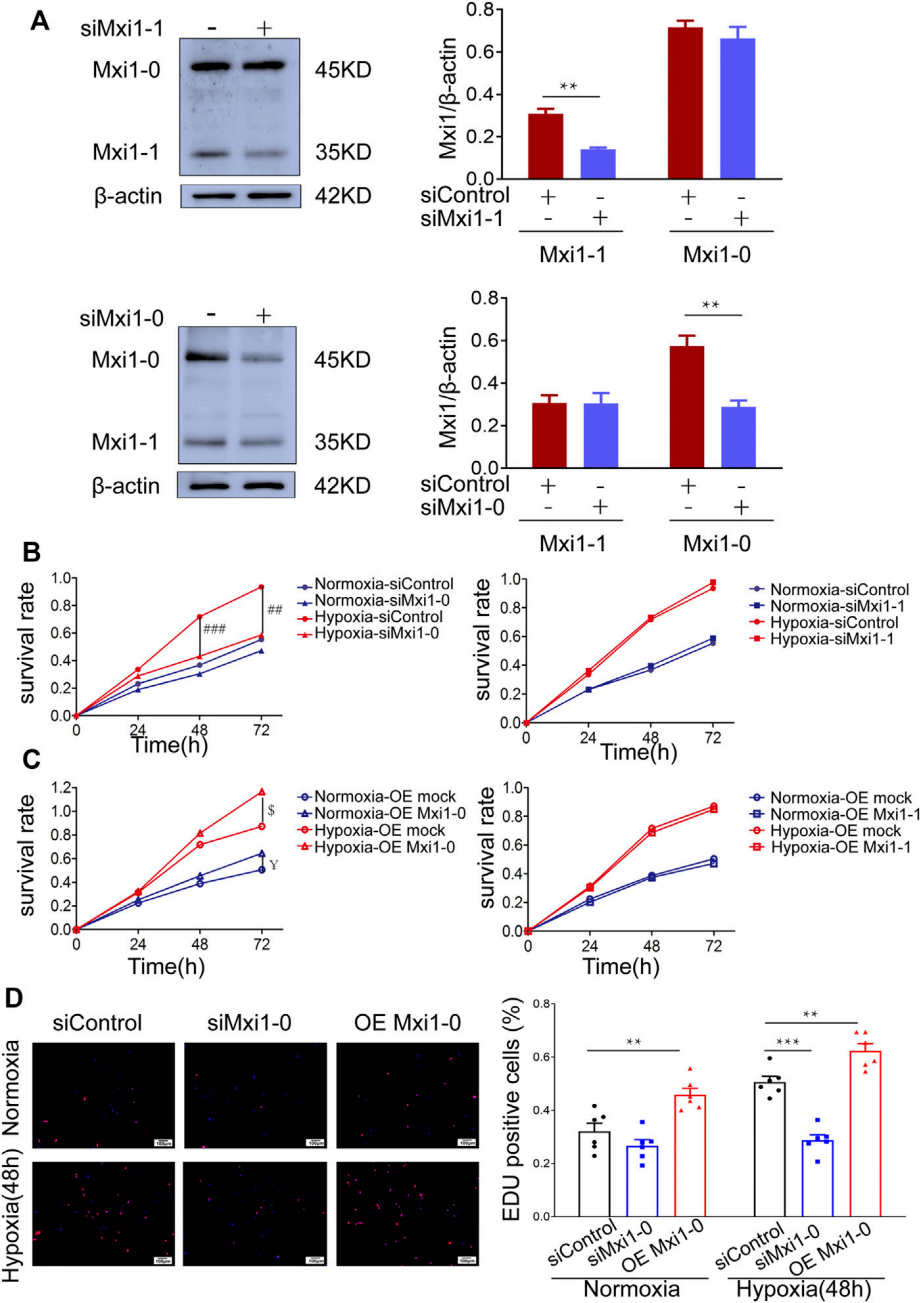
Mxi1-0 but not Mxi1-1 promotes PASMCs growth under hypoxic conditions. **(A)** PASMCs were transfected with siRNAs targeting either Mxi1-0 or Mxi1-1, and were subjected to Western blotting analyses. **(B)** PASMCs cultured in normoxic or hypoxic conditions were transfected with siRNAs targeting either Mxi1-0 or Mxi1-1, and CCK-8 assays were performed on indicated times. **(C)** PASMCs cultured in normoxic or hypoxic conditions were infected with recombinant lentiviruses to overexpress Mxi1-0 or Mxi1-1, and CCK-8 assays were performed on indicated times. **(D)** PASMCs were transfected with Mxi1-0-targeted siRNAs or infected with recombinant lentiviruses to overexpress Mxi1-0, and were cultured in normoxic or hypoxic conditions for 48 h. Cells were then subjected to immunofluorescence staining for EdU, followed by microscopy and calculation of the ratios of EdU-positive cells in three random fields. Scale bar, 100 μm. Data from three independent experiments are shown as means ± SDs. For statistical significance, *represents *p* < 0.05 compared to siControl, ^**^represents *p* < 0.01 compared to siControl, ^***^represents *p* < 0.001 compared to siControl, ^##^ represents *p* < 0.01 compared to Hypoxia-siControl, ^###^ represents *p* < 0.001 compared to Hypoxia-siControl, ^$^represents *p* < 0.05 compared to Hypoxia-OEmock, ^¥^represents *p* < 0.05 compared to Normoxia-OEmock.

Accumulating evidence has indicated that hypoxemia in the pulmonary vessels leads to increased PASMCs proliferation (Shah, 2012). To test whether Mxi1-0 plays a pathogenic role in the induction of this phenotype, we designed siRNAs that could specifically knockdown Mxi1-0 (siMxi1-0) or Mxi1-1 (siMxi1-1) (Figure 4A). While hypoxic treatment significantly promoted the growth of PASMCs, this was ablated by knockdown of Mxi1-0 but not that of Mxi1-1 (Figure4B). Conversely, overexpression of Mxi1-0 but not Mxi1-1 improved the growth rates of PASMCs exposed either to normoxia or hypoxia (Figure 4C). In line with these observations, we found that Mxi1-0 silencing in hypoxia-treated PASMCs reduced the ratios of EdU-positive cells, whereas Mxi1-0 overexpression enhanced these ratios in both normoxia- and hypoxia-exposed cells (Figure 4D), suggesting an essential role of Mxi1-0 in hypoxia-elicited mitosis of PASMCs. Collectively, these data indicate that Mxi1-0 but not Mxi1-1 drives the hyper-proliferative response in hypoxia-exposed PASMCs.

### Mxi1-0 Potentiates Pulmonary Arterial Smooth Muscle Cells Proliferation *Via* Upregulation of c-Myc

**FIGURE 5:**
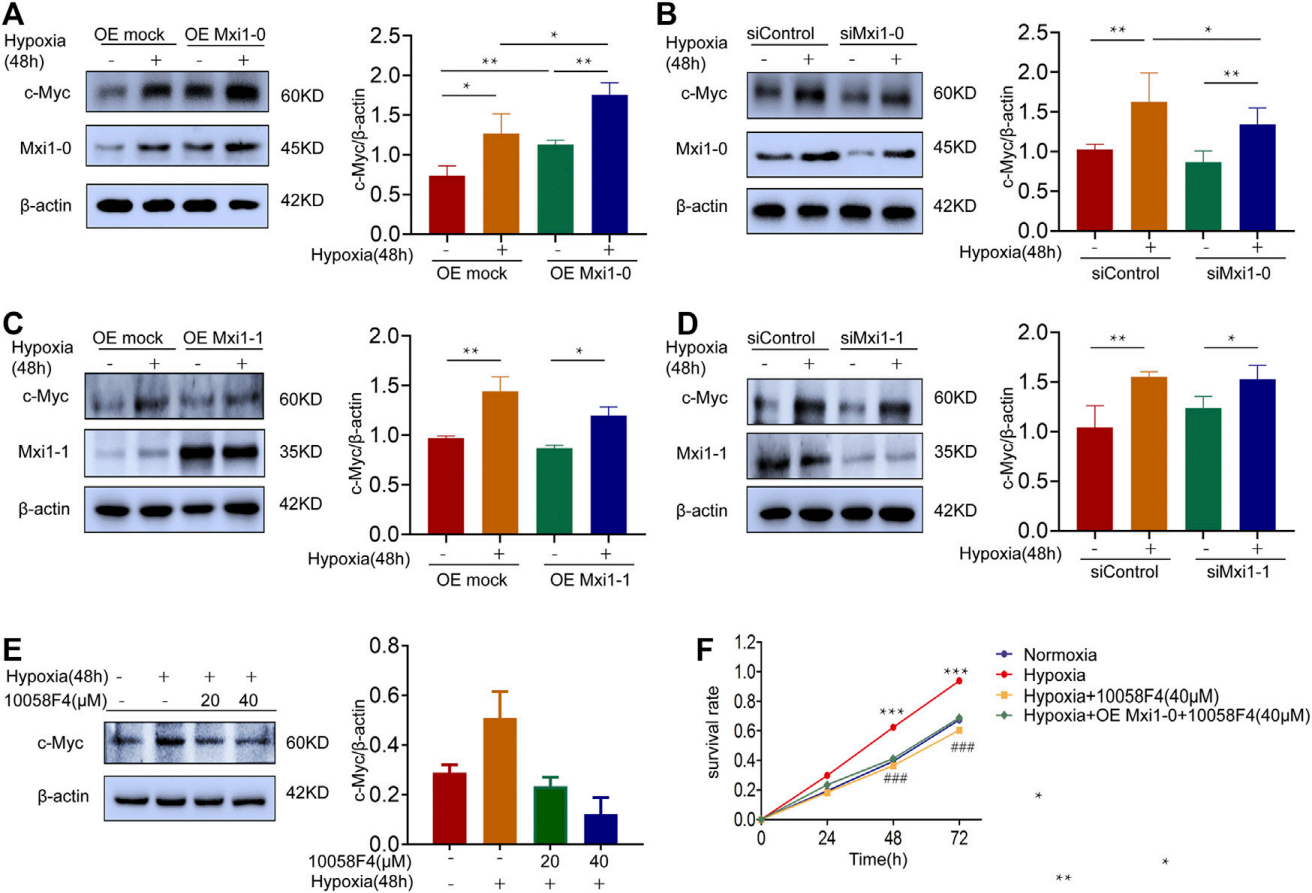
Mxi1-0 upregulates c-Myc production in hypoxic PASMCs. **(A)** PASMCs were infected with recombinant lentiviruses to overexpress Mxi1-0, and were cultured under normoxic or hypoxic conditions for 48 h. Cells were then subjected to Western blotting analysis. **(B)** PASMCs were transfected with Mxi1-0-targeted siRNAs, and were cultured under normoxic or hypoxic conditions for 48 h. Cells were then subjected to Western blotting analysis. **(C)** PASMCs were infected with recombinant lentiviruses to overexpress Mxi1-1, and were cultured under normoxic or hypoxic conditions for 48 h. Cells were then subjected to Western blotting analysis. **(D)** PASMCs were transfected with Mxi1-1-targeted siRNAs, and were cultured under normoxic or hypoxic conditions for 48 h. Cells were then subjected to Western blotting analysis. **(E)** PASMCs were cultured under normoxic or hypoxic conditions for 48 h and treated with indicated doses of 10058F4. Cells were then subjected to Western blotting analysis. **(F)** PASMCs were cultured under normoxic or hypoxic conditions, treated with 10058F4, and infected with control or Mxi1-0-overexpressing lentiviruses. Cells were then subjected to CCK-8 assays on indicated times. Data from three independent experiments are shown as means ± SDs. For statistical significance, ^*^represents *p* < 0.05 compared between two groups, ^**^represents *p* < 0.01 between two groups, ^***^represents *p* < 0.001 compared between Hypoxia and Normoxia, ^###^ represents *p* < 0.001 compared between Hypoxia and Hypoxia + 10058F4 (40 μM).

C-Myc is a proto-oncogene, which plays a key role in the regulation of cell growth and proliferation (Nesbit et al., 1999; Baluapuri et al., 2020). Previous studies have demonstrated that hypoxia leads to the high expression of c-Myc in the occurrence of pulmonary vascular diseases, and that Mxi1 is involved in the regulation of the transcription factor, c-Myc (Zervos et al., 1993; Voelkel et al., 2013). To mechanistically understand how Mxi1-0 might promote proliferation, we assessed the expression of c-Myc in hypoxic PASMCs further subjected to Mxi1-0 silencing or overexpression. As a result, hypoxic exposure increased c-Myc levels, which was further augmented by overexpression and counteracted by knockdown of Mxi1-0 (Figures 5A,B). By constrast, Mxi1-1 overexpression or knockdown failed to affect the expression of c-Myc in PASMCs cultured in normoxic or hypoxic conditions (Figures 5C,D), consistent with previous findings that Mxi1 forms a complex with Max to repress the transactivity rather than directly reduce the expression of c-Myc (Huang et al., 2018). We next probed whether Mxi1-0 promotes PASMCs growth *via* upregulation of c-Myc in hypoxic conditions. While hypoxia-induced proliferation of PASMCs was significantly impaired by a c-Myc inhibitor, 10058F4, this cannot be rescued by overexpression of Mxi1-0 (Figures 5E,F). These observations indicate that the pro-proliferative effect of Mxi1-0 is mediated by upregulation of c-Myc in hypoxic PASMCs.

### Mxi1-0 Upregulates c-Myc Through MEK/ERK Signaling in Hypoxia-Exposed Pulmonary Arterial Smooth Muscle Cells

**FIGURE 6:**
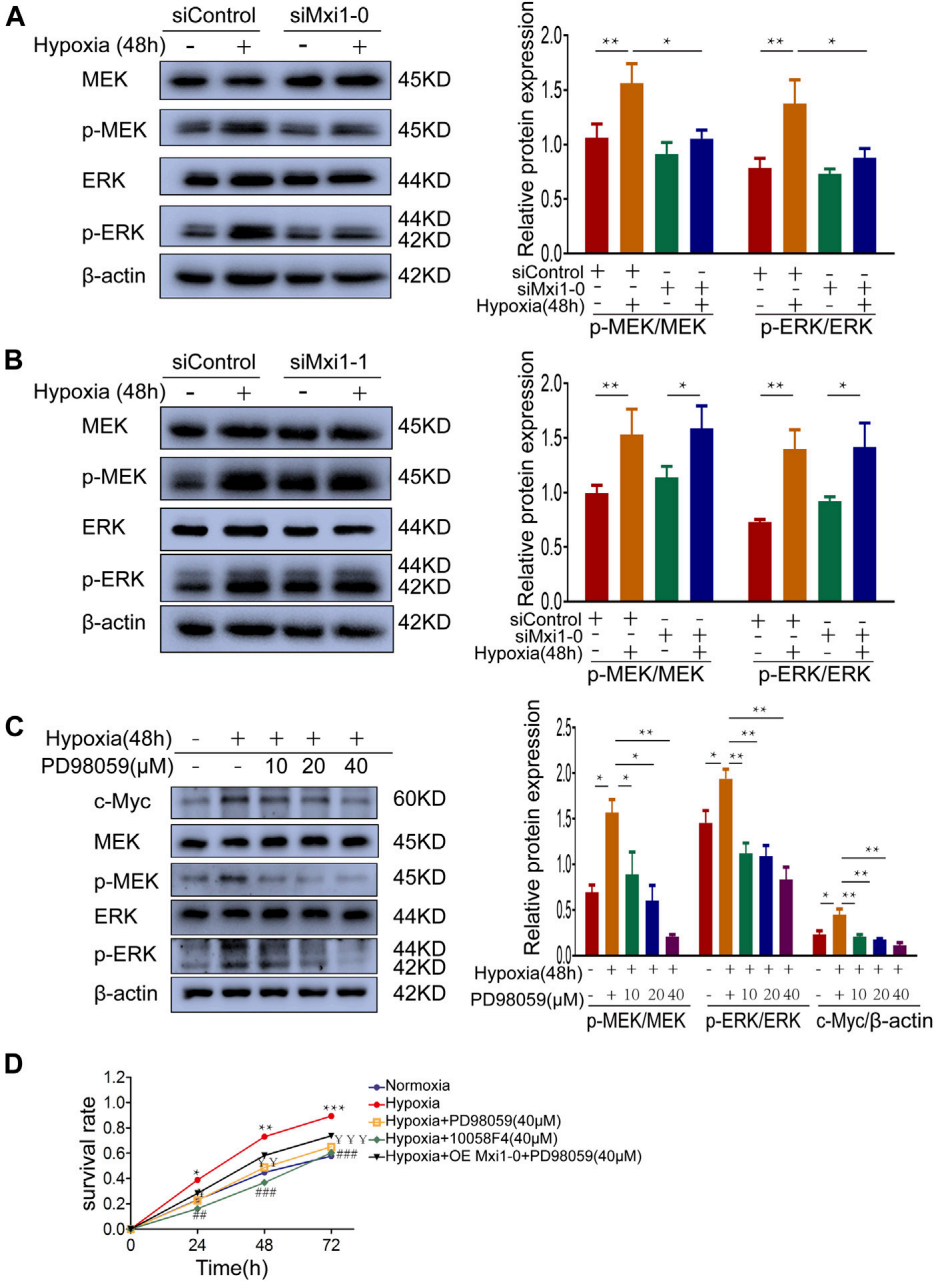
Mxi1-0 upregulates c-Myc through MEK/ERK signaling in hypoxic PASMCs. **(A,B)** PASMCs tranfected with siRNAs targeting Mxi1-0 **(A)** or Mxi1-1 **(B)** were cultured under normoxic or hypoxic conditions for 48 h. Western blotting assay was performed with the indicated antibodies. All the phospho-protein levels were measured by densitometry and normalized to that of β-actin. **(C)** PASMCs were cultured under normoxic or hypoxic conditions for 48 h and treated with different doses of PD98059, and the levels of indicated proteins were determined by Western blotting analysis and quantified by densitometry. **(D)** PASMCs were cultured under normoxic or hypoxic conditions, treated with PD98059 or 10058F4, and infected with control or Mxi1-0-overexpressing lentiviruses. Cells were then subjected to CCK-8 assays on indicated times. Data from three independent experiments are shown as means ± SDs. For statistical significance, *represents *p* < 0.05 compared between two groups or to Normoxia **(D)**, ^**^represents *p* < 0.01 compared between two groups or to Normoxia **(D)**, ^¥^represents *p* < 0.05 compared between Hypoxia and Hypoxia + PD98059 (40 μM), ^¥¥^represents *p* < 0.01 compared between Hypoxia and Hypoxia + PD98059 (40 μM),^¥¥¥^represents *p* < 0.001 compared between Hypoxia and Hypoxia + PD98059 (40 μM), ^##^ represents *p* < 0.01 compared between Hypoxia and Hypoxia+10058F4 (40 μM), ^###^represents *p* < 0.001 compared between Hypoxia and Hypoxia + 10058F4 (40 μM).

Previous studies suggested that the pathobiology of HPH was associated with the activation of MEK/ERK signaling, which was documented to promote c-Myc expression (Dang et al., 2006). In addition, bioinformatic analysis in the early stage of this study showed that the function of Mxi1-0 was enriched in Ras/MAPK signaling pathway. Hence, we examine whether Mxi1-0 upregulates c-Myc *via* MEK/ERK signaling in hypoxia-treated PASMCs. Indeed, hypoxia induced phosphorylation of MEK and ERK, which was inhibited by knockdown of Mxi1-0 but not Mxi1-1 (Figures 6A,B). Hypoxia-induced upregulation of c-Myc was counteracted by PD98059, a classical MEK antagonist, in a dose-dependent manner (Figure 6C). Similar to the inhibition of c-Myc, antagonizing MEK/ERK signaling abrogated hypoxia-induced hypoxia-elicited overgrowth of PASMCs, which was not rescured by overexpression of Mxi1-0 (Figure 6D). These data suggest that Mxi1-0 enhanced the proliferation of PASMCs through MEK/ERK-mediated upregulation of c-Myc.

**FIGURE 7:**
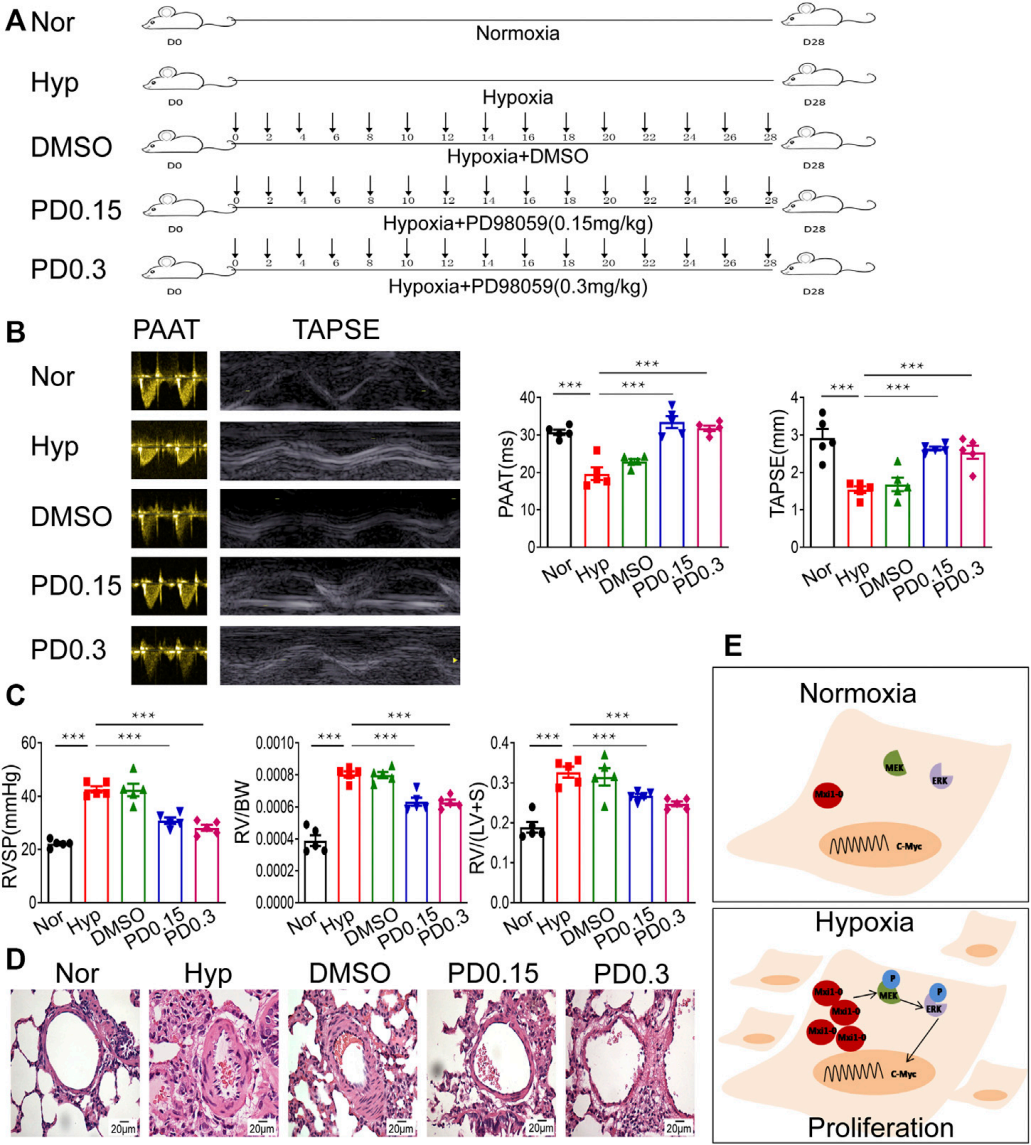
Inhibition of MEK/ERK signaling protects rats against HPH. **(A)** A rat model of hypoxic pulmonary hypertension (HPH) was generated (*n* = 5 animals per group). Rats exposed to chronic hypoxia were treated with vehicle (DMSO) or 0.15 mg/kg or 0.3 mg/kg PD98059. **(B)** Rats in all groups were subjected to echocardiography and measurement of PAATand TAPSE. **(C)** After intubation for rats described in **(A)**, RVSP was recorded, and the right ventricular hypertrophy ratio of RV/BW and RV/LV + S were calculated. **(D)** Hematoxylin and eosin staining of paraffin-fixed lung sections prepared from rats described in **(A)** was performed for morphological analysis of the pulmonary arteries. **(E)** A diagram showing that hypoxia-induced Mxi1-0 promotes PASMCs proliferation *via* MEK/ERK/c-Myc signaling in the context of HPH. Scale bar, 20 μm. Data are shown as means ± SDs. For statistical significance, ^***^represents *p <* 0.001 compared to hypoxia. PAAT, pulmonary artery acceleration time; TAPSE, tricuspid annulusplain systolic excursion; RVSP, right ventricular systolic pressure; RV, right ventricle; BW, body weight; LV + S, left ventricle plus septum.

We further explore the function of MEK/ERK signaling in HPH pathogenesis using rat models (Figure 7A). Sustained exposure of animals to hypoxia is sufficient to induce pulmonary hypertension as determined by hemodynamic (echocardiography and right heart catheter) and morphological analysis, e.g., decreased PAAT and TAPSE (Figure 7B). Treatment of rats with PD98059 significantly relieved hypertension as revealed by restored PAAT and TAPSE (Figure 7B). In addition, PD98059 treatment dramatically decreased RVSP and ameliorated RVH indices [including RV/BW ratio and RV/(LV + S) ratio], both of which are major characters of HPH (Figure 7C). HE staining revealed that PD98059 significantly improved hypoxia-induced pulmonary artery remodeling by reducing the population of PASMCs (Figure 7D). These data demonstrated that inhibition of MEK/ERK signaling protects rats against chronic hypoxia-induced HPH.

## Discussion

HPH is a serious pulmonary disorder with systemic complications that aggravate its clinical consequences. To date, no effective treatment for this life-threatening disease has advanced, which is at least partially ascribed to the lack of appropriate biomarkers (Maron et al., 2016; Thompson and Lawrie, 2017). In the present study, we found that Mxi1 was overexpressed in the pulmonary arteries of HPH patients. Immunohistochemical staining showed that Mxi1 was expressed abundantly in the medial layer mainly composed of PASMCs, which was further validated by immunofluorescence indicating that Mxi1 overlapped with α-SMA, a specific biomarker of smooth muscle cells. Although Mxi1 has been extensively studied in the context of carcinogenesis and well documented as a tumor suppressor (Zervos et al., 1993; Huang et al., 2018), this is probably the first study to determine that it is also an important player in HPH, reminiscent of multiple shared pathomechanisms between pulmonary hypertension and cancer (Negi et al., 2021).

Protein isoforms are generated from the same gene due to transcription from different promoter, alternative splicing or varied translation initiation sites (Yap and Makeyev, 2016). Although these isoforms are structurally and functionally similar, it is also common that they play distinct roles or are competitively involved in a physiological or pathological process (Li et al., 2016). The Mxi1 gene is located on chromosome 10q24-q25 (Wechsler et al., 1994), and encodes proteins of three different isoforms, Mxi1-0, Mxi1-1 and Mxi1 WR, of which Mxi1 WR is considered to have no biological function due to lack of SID. Although it is highly homologous with Mxi1-1, Mxi1-0 has an additional N-terminal sequence consisting of 92 amino acids (Engstrom et al., 2004). This novel sequence has a proline rich domain (PRD), which is responsible for the cytoplasmic localization of Mxi1-0 (Armstrong et al., 2013). In the study, we established that Mxi1-0, but not Mxi1-1, was highly expressed in PASMCs of HPH patients. Mxi1-0, but not Mxi1-1, was induced by hypoxia and plays an essential role in the proliferation of PASMCs. These observations were in contrast to Mxi1-1, which was reported to suppress cell growth through binding to Max and impairing the transcriptional activity of c-Myc (Wechsler et al., 1997; Manni et al., 2002). The unique PRD in Mxi1-0 recruits specific protein chaperones may explain why Mxi1-0 has different cellular functions from Mxi1-1 (Dugast-Darzacq et al., 2004; Hurlin and Huang, 2006). A recent study showed that the deletion of PRD converts Mxi1-0 into a potent suppressor of c-Myc, which is considered to be a key mediator of HPASMCs proliferation (Dugast-Darzacq et al., 2007; Zhang et al., 2019). Other studies suggested that although Mxi1-0 could bind to Max protein, it failed to inhibit c-Myc-dependent transcription but might promote the transcription of the proto-oncogene c-Myc (Engstrom et al., 2004; Boult et al., 2008). Nonetheless, the detailed mechanisms underlying the role of Mxi1-0 in the division of PASMCs remain to be further dissected, e.g., whether Mxi1-0 and Mxi1-1 play opposite roles in the regulation of Max-Myc interaction or whether Mxi1-0 participates in the cellular machineries responsible for degradation of c-Myc.

MAPK signaling pathway is involved in the regulation of various biological functions of cells (Uehling and Harris, 2015). The activation of this pathway licenses the expression of a large cohort of genes regulating cell proliferation, differentiation and vascular development (Lei et al., 2015). MEK/ERK signaling is a classical MAPK signal transduction pathway and serves an important regulator of pulmonary hypertension (Preston et al., 2006). Consistently, we found here that HPH-related genes were significantly enriched in MAPK signaling pathway and ERK related biological processes. Mxi1-0, as a novel regulator of HPH, potentiates PASMC proliferation through MEK/ERK-dependent upregulation of c-Myc (Figure 7E). These findings are in agreement with previous studies showing that Mxi1-0 activates MEK/ERK signaling and improved the proliferation of human umbilical vein endothelial cells (HUVECs), and that Mxi1-0 underlies hypoxia-induced vascular endothelial growth factor production by hepatic carcinoma cells (Hu et al., 2017; Wu et al., 2017). Although it is still unknown how Mxi1-0 activates MEK/ERK signaling in PASMCs, we found that inhibition of this canonical pathway significantly repressed the development of pulmonary hypertension in rats exposed to chronic hypoxia.

Collectively, we report a hitherto unrecognized crucial role of Mxi1-0 in HPH, thereby providing rationale for the applicability of Mxi1-0 and downstream signaling as candidate targets for clinical treatment and potential biomarkers for prognostic assessment of HPH.

## Data Availability

The original contributions presented in the study are included in the article/Supplementary Material, further inquiries can be directed to the corresponding author.
